# The Physical and Psychosocial Impact of Fatigue among Patients with Sjogren’s Syndrome: A Systematic Review

**DOI:** 10.3390/jcm13061537

**Published:** 2024-03-07

**Authors:** Denise-Ani Mardale, Daniela Opriș-Belinski, Violeta Bojincă, Mihai Bojincă, Diana Mazilu, Emilia Păsăran, Cristina Nițăa, Laura Groșeanu, Florian Berghea, Andra-Rodica Bălănescu

**Affiliations:** 1 Faculty of Medicine,‘Carol Davila’ University of Medicine and Pharmacy, 050474 Bucharest, Romania; daniela.opris@umfcd.ro (D.O.-B.); violeta.bojinca@umfcd.ro (V.B.); mihai.bojinca@umfcd.ro (M.B.); diana.mazilu@umfcd.ro (D.M.); emilia-daniela.pasaran@drd.umfcd.ro (E.P.); elena-cristina.nita@rez.umfcd.ro (C.N.); maria.groseanu@umfcd.ro (L.G.); florian.berghea@umfcd.ro (F.B.); andra.balanescu@umfcd.ro (A.-R.B.); 2 Department of Internal Medicine and Rheumatology, ’Sf. Maria’ Clinical Hospital, 011192 Bucharest, Romania; 3 Department of Internal Medicine and Rheumatology, ‘Dr. Ion Cantacuzino’ Hospital, 020475 Bucharest, Romania

**Keywords:** primary Sjogren’s syndrome, fatigue, predictive factors, sleep disturbance, cognitive concerns

## Abstract

**Background:** Primary Sjögren’s syndrome (pSS) is a complex autoimmune disorder characterized by organ-specific symptoms in the salivary and lacrimal glands, as well as systemic manifestations. Fatigue, a prominent aspect, significantly influences the overall quality of life for individuals with pSS. **Methods:** This review seeks to evaluate the impact of fatigue by exploring its consequences, potential causes, and effects on physical and psychological well-being, while also investigating its management strategies. Following the “Preferred Reporting Items for Systematic Reviews and Meta-Analyses (PRISMA)” guidelines, our systematic literature review involved a five-step algorithm. Initially identifying 78 articles in reputable international medical databases, we applied eligibility criteria and removed duplicates, resulting in 19 articles for qualitative synthesis. **Results:** This review delves into the predictive factors for heightened fatigue in pSS, encompassing rheumatoid factor levels, erythrocyte sedimentation rate, and immunoglobulin G levels. Sleep disturbances, specifically nighttime pain and nocturia, emerged as determinants of persistent daytime fatigue. Cognitive impairment in pSS involves deteriorations in global memory, executive functioning, and attentional resources. Furthermore, functional limitations in pSS impact patients’ quality of life. **Conclusions:** The significance of fatigue in pSS, its consequences, and profound influence on the quality of life necessitate further research for a more comprehensive understanding of this complex issue.

## 1. Introduction

Primary Sjögren’s syndrome, a prevalent systemic autoimmune disorder characterized by exocrinopathy, presents a spectrum of symptoms and diverse disease severity, with pronounced dryness in the mouth and eyes and persistent fatigue being prominent [1,2,3].

This condition primarily affects women in their fifth or sixth decades of life, and its pleomorphic clinical presentation, involving various organ systems, underscores its intricate impact on individuals, resulting in varying global incidence and prevalence rates, as revealed in an epidemiological review [4].

This autoimmune exocrinopathy exhibits a spectrum extending from organ-specific to systemic autoimmunity, with systemic symptoms affecting 50–60% of patients associated with primary Sjogren’s syndrome (pSS) [5], impacting various body systems such as the skin, joints, pulmonary, cardiovascular, renal, hematological, and nervous systems. These extra-glandular manifestations, including persistent fatigue, widespread joint and muscular pain, and cognitive impairments, not always aligning with serologic indicators, significantly contribute to a diminished quality of life [6].

Fatigue, encompassing physical and mental components, refers to tiredness, weakness, lack of energy, and concentration difficulties [7]. Prevalence estimates indicate 20.4% in the general population suffer from fatigue lasting <6 months, 10.1% suffer from chronic fatigue [8], and 60–70% of patients with autoimmune diseases also suffer from fatigue. Despite various influencing factors, overall health status remains a significant contributor to fatigue occurrence [9,10]. The impact of fatigue has been extensively explored across various medical conditions, revealing significant economic consequences, with a two-week prevalence of 38% among U.S. workers, resulting in an estimated annual cost exceeding 136 billion USD due to lost productive work time [11].

In rheumatic diseases, including primary Sjogren’s syndrome, fatigue emerges as a substantial predictor of work-related dysfunction and overall health status, prioritized alongside well-being but surpassing joint symptoms [12,13,14,15,16,17]. Recent studies underscore the critical role of fatigue in determining the quality of life for individuals with Sjogren’s syndrome [18,19,20], emphasizing the need for a comprehensive assessment using multiple evaluation scales to understand its true dimensions over time [21,22,23].

This review aims to comprehensively explore the multifaceted nature of fatigue in primary Sjögren’s Syndrome, addressing its impact on both physical and psychosocial aspects and the overall quality of life. This study also delves into potential causes, including inflammatory and immunological conditions, and evaluates various management and therapy alternatives to improve patients’ well-being and mitigate the detrimental effects of fatigue, with the overarching goal of enhancing diagnosis, treatment, and the overall quality of life for individuals affected by this complex illness.

## 2. Materials and Methods

**Figure 1:**
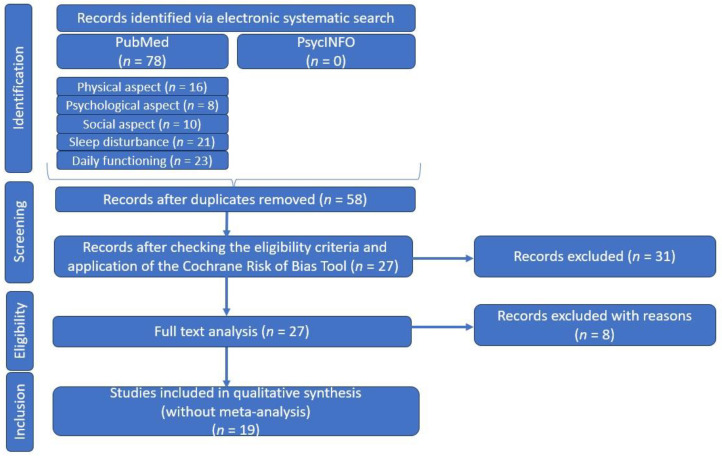
PRSIMA diagram—customized to our systematic literature review.

As the relationship between primary Sjogren Syndrome, fatigue, and quality of life is a topic of ongoing interest and debate, we have undertaken a comprehensive systematic literature review, following the principles of the Preferred Reporting Items for Systematic Reviews and Meta-Analyses (PRISMA: we did not register for this research), in order to provide an overview of the existing research on the physical and psychosocial impacts of fatigue in pSS (Figure 1).

Given that fatigue in pSS is a significant and often overlooked problem that can have substantial consequences on daily life, it is important to have some predictive factors. This can help us with the early recognition of individuals who may be at risk of experiencing fatigue and understanding the factors that contribute to this will help in determining some strategies for its treatment. In this context, some studies have been carried out to pinpoint predictive factors in individuals with pSS.

The first step involved searching for relevant articles in reputable international medical databases such as the National Center for Biotechnology Information (NCBI)/PubMed and in PsycINFO, imposing any time restrictions. In our research, we used specific keyword combinations/“syntaxes”, as follows: (physical aspect) AND (fatigue) AND (Sjogren’s syndrome OR Sjogren’s disease OR Sicca), (psychological aspects) AND (fatigue) AND (Sjogren’s syndrome OR Sjogren’s disease OR Sicca), (social aspects) AND (fatigue) AND (Sjogren’s syndrome OR Sjogren’s disease OR Sicca), (sleep disturbance) AND (fatigue) AND (Sjogren’s syndrome OR Sjogren’s disease OR Sicca), or (daily functioning) AND (fatigue) AND (Sjogren’s syndrome OR Sjogren’s disease OR Sicca) (Table 1).

After the first search stage, a total of 78 articles were returned and in the second stage, after removal of duplicates, there remained 58 articles that completed our list.

In our search, we selected articles whose type of study was classified as “original research” or “clinical trials”, without imposing a time limit, and study participants were patients with pSS, diagnosed according to international criteria [24,25]. Although no time limit was selected, all articles were published from 1990 onwards, in most cases. We considered only articles in English and excluded articles whose type of study was categorized as reviews, case reports, or comments, or those with only abstracts available. Incomplete or unextractable data were also excluded, along with articles including patients with secondary Sjogren’s syndrome, a pediatric population, or animals.

After verifying these eligibility criteria, 39 articles were eliminated, leaving us with 19 articles for qualitative synthesis in the next step.

## 3. Results

Table 2 contains the selected articles after our systematic literature review. This presents the characteristics of the experimental groups along with a designated “Control lot”. The term “Control lot” refers to a specific group within the study that serves as the control group for comparative analysis. Data from this control group provide a baseline against which the results of the experimental groups are evaluated.

### 3.1. Predictors of Fatigue

Acknowledging the established connection between chronic fatigue and depression, Segal et al. performed a study involving patients with pSS. Their objective was to assess how various behavioral, cognitive, and clinical factors contribute differentially to fatigue. Subdividing fatigue into mental and somatic, it was observed that somatic fatigue is more pain-weighted, while mental fatigue increased with depression. There was a connection between extreme fatigue and depression among the patients, although fatigue was not identified as the primary causative factor for depression. Another conclusion drawn from the study is that individuals who perceive themselves as having little influence or control over their condition are more prone to experiencing fatigue and depression [44].

Aligned with the aforementioned findings, another study by Lendrem et al. discovered that individuals diagnosed with primary Sjögren’s syndrome (pSS) manifest significantly diminished utility values. The main predictive factors identified were pain and depression. The impact of fatigue and dryness symptoms on overall EQ-5D scale utility values showed relatively modest results. This implies that focusing on pain and depression may bring about a more substantial enhancement in the quality of life for patients with pSS compared to addressing fatigue and dryness [42].

Looking at paraclinical evaluations, research investigations have delved into examining the potential relationship between the presence of antibodies and the extent of fatigue experienced by individuals. In the aforementioned study, it was observed that individuals experiencing fatigue had lower levels of rheumatoid factor (RF), erythrocyte sedimentation rate (ESR), immunoglobulin G (IgG) levels, absolute lymphocyte count, and antinuclear antibody (ANA) positivity, compared to those without fatigue. Additionally, there is some evidence suggesting that among patients with positive anti-Ro and anti-La antibodies, there is a correlation with fatigue but not with depression [44], while other research conducted by Theander et al. has found no link between anti-Ro and anti-La antibodies and fatigue [26].

In conclusion, the identification of subclinical depressive symptoms and a thorough understanding of the factors contributing to depression in individuals with primary Sjögren’s syndrome (pSS) are essential for its effective management [44]. Furthermore, there is a requirement for additional longitudinal data to clarify the relationship between fatigue and immunologic disease activity in pSS [44], along with the development of more comprehensive instruments that capture the full impact of symptoms on the generic health-related quality of life (HRQoL) [42].

### 3.2. Fatigue and Sleep Disorder

There are complex and convincingly bidirectional relationships between sleep and fatigue on the one hand, and other variables like pain or psychological distress on the other. Moreover, daytime sleepiness is an important problem of primary Sjogren’s syndrome [26,34,36].

The higher level of fatigue among patients with pSS compared to healthy individuals was observed in a study conducted by Theander et al. It was also noted that these patients frequently experience daytime sleepiness and sleep disturbances, which correlate with their fatigue. Two main factors influencing fatigue were identified, as follows: nighttime pain and nocturia. Additionally, nighttime awakenings due to dryness symptoms significantly contribute to inadequate sleep. Thus, it has been found that the discomfort keeping patients awake contributes to the onset of depression and anxiety [26].

These findings underscore the possibility that treating and reducing nighttime pain and nocturia may lead to a reduction in anxiety and depression, subsequently alleviating fatigue [26].

In addition to the nighttime pain and nocturia caused by an increased water intake due to dryness experienced by pSS patients, a study conducted by Goulabchand et al. shows that these patients have an increased likelihood of experiencing sleep disturbances. Therefore, this reduced sleep quality is highlighted by excessive daytime sleepiness and a risk of sleep apnea and insomnia, emphasizing the influence of health-related issues on the level of fatigue [34]. In line with these results, a study conducted by Priori et al. observed that these individuals undergo changes in their perception of sleep quality, disruptions in their daily routines, and an increased level of disability [36].

Another significant determinant of sleep quality was represented by nocturnal respiratory issues, as studied by Yeh et al. Thus, among pSS patients, specific sleep disorders were identified, such as reduced sleep efficiency, an increased frequency of nighttime awakenings, and a heightened incidence of hypopnea events. Hence, the hypothesis was formulated that the existence of hypopnea events could be linked to obstructive mechanisms and a potential disturbance in the regulation of the central nervous system [45]. To continue on this topic, the results of a study led by Goulabchand et al. suggests that using the Berlin questionnaire, Epworth sleepiness scale, and Insomnia severity index in daily practice could help identify candidate patients for polysomnography [34].

Sleep quality and fatigue can be influenced not only by physical factors but also by psychological issues like depression. In a research effort led by Chung et al., which focused on South Korean patients with pSS, the findings indicated that 46.1% of these individuals reported subpar sleep quality. Interestingly, even among pSS patients who were not experiencing depression, 32.4% encountered sleep challenges. It was evident that patients grappling with sleep problems also displayed more severe glandular and extra-glandular symptoms when compared to those who had a higher quality of sleep [37].

**Figure 2:**
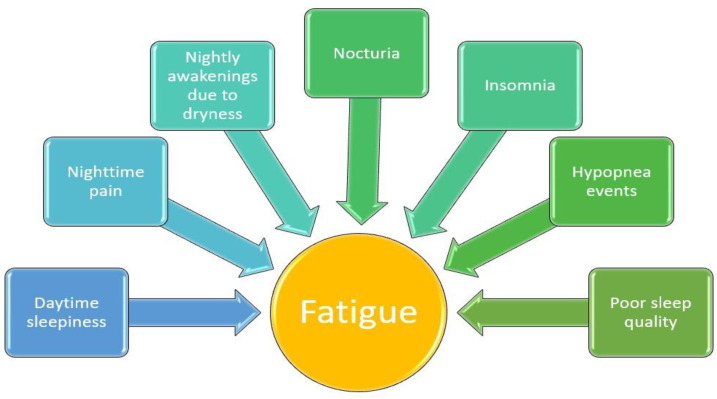
The correlation between different sleep-related symptoms and fatigue among patients with pSS.

Figure 2 provides a summary of the connection between fatigue and various sleep-related issues in individuals diagnosed with Sjogren’s syndrome.

Dardin et al. conducted a distinct study revealing a significant connection between sleep disturbances and disease activity, employing actigraphy as a tool to assess sleep–wake patterns [35]. The heightened physical and mental fatigue, especially daytime fatigue, experienced by those with pSS leads to an increased demand for healthcare resources and is associated with work-related disabilities and earlier retirement, all of which are impacted by psychological and social factors [35,46].

The study by Priori et al. establishes a novel link between sleep quality in pSS and disease activity and damage, finding that sleep disturbances are not influenced by these factors, and reveals no correlations between reduced sleep quality and patients’ immunological profiles [36].

Additionally, a study conducted by Chung et al. noticed an independent association between poor sleep quality in pSS patients and the ESSPRI [47]. It was also suggested that the ESSDAI might be underestimated in poor sleepers due to the potential negative association between poor sleep quality and parameters such as white blood cell (WBC) or neutrophil counts, as well as IgG levels. Patients with pSS experiencing poor sleep reported more severe arthralgia or myalgia and a greater degree of fatigue [37].

### 3.3. Daily Pattern of Fatigue

Considering that fatigue represents a constantly evolving condition, adapting to a range of physiological and situational factors, and exhibiting fluctuations throughout the day, Godaert et al. performed a study that examined some aspects of fatigue in the daily environment of patients with pSS and systemic lupus erythematosus (LES). This fatigue was analyzed at 15, 30, and 45 min after awakening and it was perceived that patients with pSS showed slightly increasing fatigue, while patients with LES showed decreasing fatigue [27].

Powell et al. have proposed a hypothesis suggesting that the heightened fatigue felt from the morning is linked to an increased secretion of cortisol, thereby implicating the functioning of the hypothalamic–pituitary–adrenal (HPA) axis. This axis may undergo changes in individuals with pSS [48].

The most prevalent non-exocrine symptom is extreme, impairing fatigue, which has been explored in several research articles covering the pathophysiology, etiology, evaluation, and approaches to the treatment of fatigue, but Barendregt et al. observed a significant negative correlation between the level of fatigue and noradrenaline values [29].

This observation suggests a potential connection with subtle, subclinical irregularities in the autonomic nervous system, which could be contributing to the experience of fatigue [29].

The fatigue pattern was analyzed in a study conducted by Godaert et al., which observed that in both healthy individuals and those with SLE, fatigue initially declined before increasing. However, patients with pSS exhibited a somewhat contrasting trend, at least during the early part of the day, with variations in general fatigue, physical fatigue, and reduced activity throughout the day [27].

As suggested in a study by Andersson et al., individuals with pSS often identify fatigue as the primary psychological stressor associated with their health [49]. Building on this idea, Goodchild et al. conducted a study to investigate the diurnal pattern of fatigue and its correlation with nighttime discomfort and sleep disturbances in both primary Sjögren’s syndrome (pSS) and rheumatoid arthritis (RA) patients. The findings indicated a progressive increase in the severity of both somatic and mental fatigue throughout the day in both pSS and RA patients. While arthralgia was reported as the most severe discomfort symptom in RA, pSS patients experienced a more severe discomfort related to oral sicca compared to RA patients. Actigraphy was used to examine sleep disturbance and the results showed that women with pSS slept less efficiently than women with RA [38].

The study confirmed the persistent presence of fatigue as a consistent issue throughout the day for individuals with both pSS and RA. Moreover, it revealed a connection between an increased evening discomfort and heightened fatigue on the subsequent day in women suffering from pSS or RA. Notably, this relationship was found to be statistically influenced by poor sleep, meaning that both evening discomfort and disrupted sleep collectively contributed to exacerbating fatigue on the following day [38].

Also, Van Oers et al. conducted a study focused on examining the variation in fatigue levels throughout the day. Their objective was to assess fatigue at eight distinct time points across two consecutive weekdays in patients with pSS, RA, and SLE, as well as a healthy control group, all within a natural environment. The study’s findings indicated that during the initial hour after waking, fatigue levels decreased in patients with SLE and RA, but either increased or remained unchanged in patients with pSS. This implies that the most effective way to deal with fatigue at this point might include interventions that focus on cognition and behavior [39].

### 3.4. The Functional Status and Fatigue

Considering the multitude of methods and scales available for assessing fatigue, this article provides a condensed summary (Table 3) that includes the primary fatigue assessment scales for a comprehensive analysis.

#### 3.4.1. Physical Aspects and Fatigue

The relationship between physical activity and its consequences on fatigue among patients with pSS has been debated in the literature in recent years. Physical activity is a crucial aspect in maintaining health, as it plays a vital role in preserving one’s well-being. This is underscored by its well-documented benefits for brain health, weight management, reducing the risk of disease, muscle and bone strengthening, and enhancing one’s capacity to handle daily tasks [33].

Nevertheless, Hacket et al. outlined that individuals diagnosed with pSS demonstrate a reduced capacity to perform a variety of daily tasks compared to age- and gender-matched individuals in good health [6].

In the previously mentioned study, it was observed that the functional limitations of pSS patients are linked to several clinical characteristics of their condition, albeit not necessarily all of them. These limitations also correspond to a decrease in the health-related quality of life, as a significant correlation was found between the quality of life and some physical symptoms such as pain or dryness, and the somatic aspects such as fatigue and depression. They also described an association between the CRP (C-reactive protein) and functional disability, indicating the contribution of inflammatory status to functional capacity [6].

The study conducted by Strömbeck et al. investigated the association between fatigue and physical, functional, and mental aspects. The results of this study revealed a striking contrast with the general population regarding functional disability, indicating that women diagnosed with pSS exhibited a reduced functional capability [30].

While there is no significant increase in the prevalence of a sedentary lifestyle among people with pSS, Ng et al. demonstrated that physical activity levels are significantly lower among patients with pSS. These lower activity levels were independently associated with symptoms of depression and daytime sleepiness, ultimately resulting in mental and physical fatigue [28].

Additionally, in the aforementioned study, it was observed that depression may be an independent predictor of a decreased level of physical activity. Similarly, it is important to note that, unlike men, women typically engage in less physical activity [28].

The relationship between depression, fatigue, and physical activity was analyzed in a study conducted by Strömbeck et al. Thus, the reason why women with pSS experience increased depression could be explained by the idea of a limited engagement in physical activity, but more research is required to validate this hypothesis and to conclude that the symptoms of fatigue can be mitigated by increasing physical activity. It has been demonstrated that the women with pSS had a reduction in aerobic capacity, joint mobility in the upper extremities, muscle function, and standing balance compared with the general population. This group experienced a greater effort than the control group when performing the bicycle test and had a significantly lower strength and endurance of the knee flexors. Age, aerobic capability, depression, and functional impairment all contribute to the experienced exhaustion [30].

This led to the conclusion that aerobic training as part of a physical exercise strategy could decrease fatigue [30].

Another important physical complaint of patients with pSS is vaginal dryness. A study conducted by Al-Ezzi et al. demonstrated that sexual dysfunction was twice as prevalent in pSS patients. Impaired sexual function negatively affected the social aspect of the quality of life for patients with pSS, without significant influence on the physical or psychological domains. The primary concerns for patients were related to oral dryness and fatigue, as opposed to issues associated with vaginal dryness or joint problems. Sexual dysfunction in pSS patients is a multi-dimensional issue that may arise from factors like fatigue and joint pain, rather than being solely attributed to vaginal dryness [50].

#### 3.4.2. Cognitive Aspects and Fatigue

It is known that cognitive impairment, often appearing as difficulties related to memory, attention, or information processing, can be both distressing for patients and a complex puzzle for healthcare providers, due to the numerous potential underlying reasons for these symptoms [51].

Patients with pSS were observed to demonstrate cognitive dysfunction, which manifested as impairments in attention, information processing speed, executive functions, verbal memory, visual memory, and visual–spatial perception, as well as lower scores for motor reaction time [32,40,52,53,54].

In a study led by Barendregt et al., it was observed that patients with pSS often struggle with severe fatigue. However, physical fatigue, general fatigue, and reduced activity may also be related to the disease. Some aspects of fatigue, such as mental fatigue and reduced motivation, can most likely be attributed to depressive symptoms [29].

A key reference study is that of Segal et al., whose aim is to investigate the intricate relationship between the perception of cognitive dysfunction, the presence of depression, and the existence of objective cognitive impairment in patients with pSS. It is important to note that these patients have no prior history of central nervous system disorders beyond their cognitive concerns. This relationship sheds light on the interplay of cognitive issues, emotional well-being, and clinical cognitive performance within this specific patient group [32].

This study demonstrated that depression and verbal memory function acted as separate and distinct predictors of cognitive symptoms. This suggests that both depression and verbal memory have their own unique influence on the development and manifestation of cognitive issues [32].

This concept is further reinforced by a study conducted by Koçer et al. that identified challenges when assessing cognition with regards to attention, information processing speed, and verbal learning, as well as both immediate and long-term verbal memory and visual–spatial perception in patients with pSS. This implies that individuals undergoing these assessments exhibited difficulties in maintaining their focus, processing information quickly, acquiring verbal knowledge and retaining it in both the short-term and long-term, and in perceiving spatial information visually [40].

The previously mentioned study noted that within the pSS group, significant impairments were observed in physical function, bodily pain, general health, vitality, and emotional role functioning in terms of daily living activities. Additionally, all the assessments related to depression, fatigue severity, health state, and the quality of life demonstrated a significant positive correlation with each other [40].

Subtle cognitive deficiencies, especially those related to verbal reasoning, cannot be solely attributed to depression [32]. However, it is important to acknowledge that both depression and chronic pain can significantly complicate the assessment of cognitive function. While these factors can influence cognitive performance, it is essential to recognize that the cognitive deficits observed, particularly in verbal reasoning, cannot be solely pinned on depression. These deficits may be the result of a more complex interplay of factors [40].

Patients diagnosed with pSS predominantly reported issues related to their memory. Notably, in a study conducted by Goulabchand et al., more than 50% of cases showed abnormal scores in cognitive assessments, particularly related to overall memory, executive functions, and instrumental functions [34]. Primary Sjogren’s syndrome patients with cognitive concerns display measurable impairments in global memory, executive functioning, and attentional resources [34].

**Figure 3:**
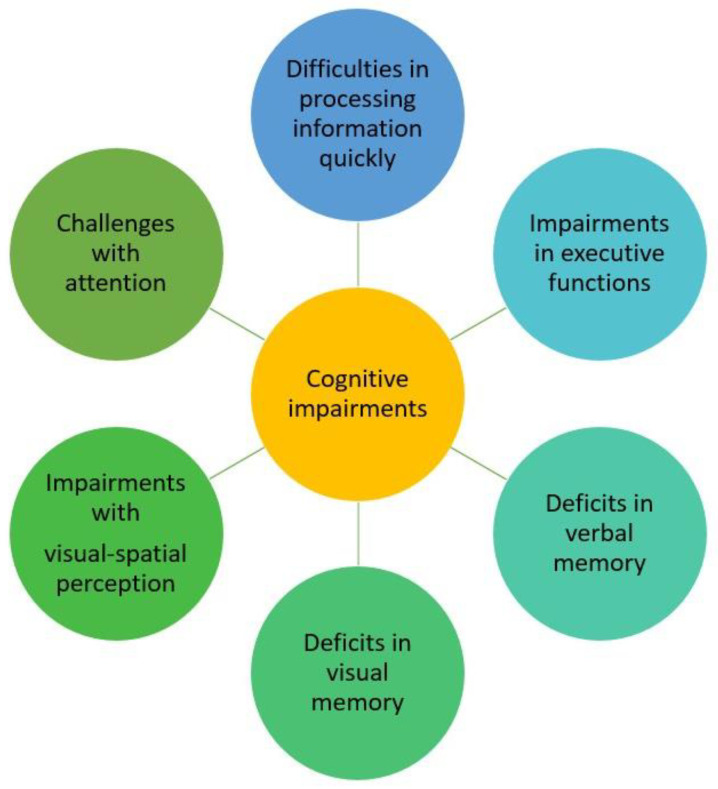
The range of cognitive impairment types seen in pSS patients.

A summary of the variation in the types of cognitive impairment observed in pSS patients, based on previous studies, can be seen in Figure 3.

Most of the studies included in this systematic review have expressed a strong interest in examining cognitive impairment in patients with pSS. Consequently, we have created a summary of the scales utilized for assessing cognition (Table 4). Each symptom presented in this table has the potential to establish a connection to cognitive impairment, much like the association observed between fatigue and low-grade inflammation.

For a better assessment of patients predisposed to cognitive impairment, it was suggested that incorporating Prof-M in clinical practice could serve as a valuable tool for identifying individuals who could potentially gain from comprehensive neuropsychological assessments. This means that Prof-M may effectively assist in the targeted identification of patients who would most benefit from in-depth cognitive evaluations [32]. Utilization of comprehensive neuropsychological assessments can effectively identify both subclinical and clinical cognitive dysfunction in individuals with pSS. Specifically, tests such as the Clock Drawing test, PASAT, and AVLT prove to be highly valuable tools for evaluating attention, information processing speed, and executive functions, as well as both short-term and long-term verbal memory in pSS patients [40]. 

### 3.5. New Perspectives in Treating Fatigue

The underlying mechanism of fatigue remains unclear, but it is suggested that immune dysregulation may play a role [13,26,55]. Studies in both humans and mice have demonstrated that the stimulation of the vague nerve (VNS), using implanted electrodes, can influence the immune process through the cholinergic anti-inflammatory reflex, which involves the vague nerve’s connection to the spleen. It is established that acetylcholine is involved in this inflammatory reflex within the spleen, leading to the suppression of TNF-α, IL-1β, and IL-18 production in macrophages when stimulated with lipopolysaccharide (LPS) [13,55,56].

Building upon these concepts, Tarn et al. conducted a study in which they measured the systemic cytokine response to lipopolysaccharide (LPS) before and after VNS. In this research, IP-10 was found to be detectable in the majority of subjects without LPS stimulation, whereas IFN γ, IL12-p70, IFNα, and IL-10 were undetectable in both stimulated and unstimulated conditions. MIP1a, IL-1ß, TNF-a, IL-6, and IP-10 were detectable after stimulation [41].

The results revealed a positive correlation between baseline T-cell and NK-cell numbers and improvements in fatigue scores. These findings suggest that non-invasive VNS may improve the subjective symptoms of fatigue and sleepiness [41].

Starting from the premise developed in a study on lupus erythematosus systemic patients by Derksen et al. that stated that decreased levels of dehydroepiandrosterone (DHEA) and its sulfate ester (DHEAS) might impact fatigue and functionality [57], Hartkamp et al. conducted a comparative study between the levels of DHEAS in women with pSS and healthy control subjects.

The study findings revealed that females with pSS reported higher levels of fatigue and depressed mood, as well as lower levels of well-being and impaired physical functioning, in comparison to the control group. Furthermore, these patients exhibited lower serum DHEAS and hemoglobin levels, elevated ESR and serum-IgG levels, and increased occurrences of dryness and pain [43].

Interestingly, fatigue, depressed mood, well-being, and physical functioning did not show significant correlations with laboratory measurements or demographic factors. However, a greater physical impairment was associated with an increased number of tender points. Additionally, both more pronounced physical dysfunction and fatigue were linked to reduced ocular dryness. These findings do not indicate that DHEA plays a role in the fatigue, well-being, and physical functioning of women with pSS [43].

The treatment goal is to address fatigue, enhance well-being, and improve physical functioning using behavioral strategies such as lifestyle adjustments, cognitive behavioral techniques, physical exercise, and interventions for improving sleep hygiene [43].

An examination carried out by Hacket et al. through focus groups, encompassing individuals diagnosed with confirmed primary Sjogren’s syndrome (pSS), unveiled a range of sleep disturbances. The results exposed an intersection between the sleep and fatigue symptoms among the participants, even though there was a clear difference between the fatigue they encountered and mere “tiredness”. The study participants were noted for actively utilizing diverse strategies to confront and navigate their sleep difficulties. Cognitive behavioral therapy emerged as a highly regarded intervention, and is considered acceptable when supported by a justified rationale and specifically tailored for individuals with pSS [6].

**Figure 4:**
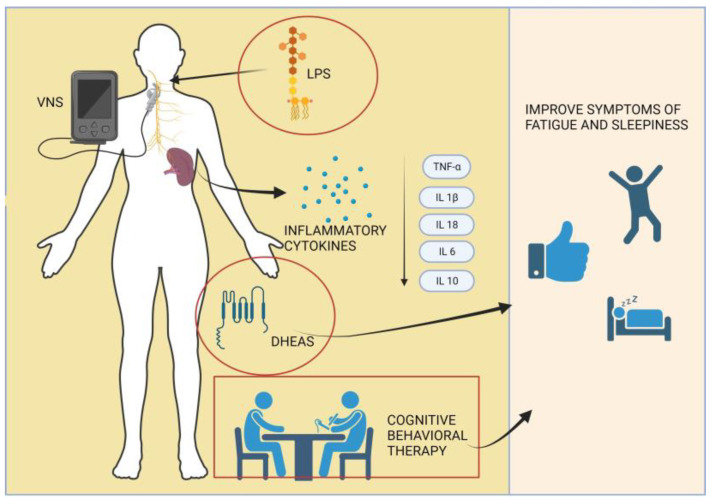
Diverse pathways underlying an innovative approach to managing fatigue in primary Sjögren’s syndrome patients.

In summary, the complex interplay of immune mechanisms, neurostimulation, and hormonal factors underscores the multifaceted nature of fatigue in primary Sjögren’s syndrome (pSS), with ongoing research offering potential insights into targeted therapeutic interventions, as illustrated in Figure 4.

## 4. Discussion

Fatigue is a topic of great significance, not just within the general population but particularly within the context of autoimmune diseases. However, the exact mechanism responsible for fatigue remains only partially elucidated. In recent decades, it has attracted research attention due to its substantial influence on the overall quality of life for the affected individuals.

Clinical trials in patients with pSS have consistently shown a robust association between depression and fatigue. Notably, somatic fatigue is closely linked to pain, while mental fatigue is correlated with depression [44].

The connection between the somatic and mental aspects of fatigue can be elucidated by considering the interplay of biological, psychological, and behavioral factors. This intricate relationship involves disruptions in neurotransmitters, specifically serotonin, dopamine, and norepinephrine, which play implicit roles in both fatigue and depression [58]. Additionally, hormonal changes contribute to this connection, primarily through the involvement of the hypothalamic–pituitary–adrenal (HPA) axis. A prolonged exposure to chronic stress can lead to the dysregulation of the HPA and result in elevated cortisol levels at inappropriate times. These dysregulations may play a role in morning fatigue for patients with primary Sjogren’s syndrome [59].

Neurotransmitters, like serotonin, dopamine, and norepinephrine, act as brain messengers, impacting mood, energy levels, and overall well-being. Imbalances in these chemicals are linked to symptoms of fatigue and depression, shaping how individuals perceive and manage both physical and mental fatigue.

In understanding the connection between fatigue and depression concerning psychological factors, it is important to highlight cognitive effects and negative thought patterns. Consequently, persistent fatigue leads to cognitive impairment, difficulties in concentration, and memory issues, fostering feelings of frustration and hopelessness commonly associated with depression. The prolonged state of fatigue also often results in diminished motivation and energy levels, giving rise to negative thoughts and a sense of helplessness, thereby contributing to the onset or intensification of depressive symptoms. Furthermore, a novel concept termed “helplessness” has been highlighted, underscoring its relevance within the context of psychosocial factors and pain among individuals with pSS. This concept significantly enhances our understanding of the factors contributing to the emotional experience of these patients [44].

Another significant concept revolved around identifying factors that can predict fatigue. While the available data may not be entirely definitive, it is reasonable to assert that certain factors show a strong association with elevated fatigue levels in pSS, including rheumatoid factor, erythrocyte sedimentation rate (ESR), Ig G levels, absolute lymphocyte count, and antinuclear antibody (ANA) positivity. Nevertheless, the impact of seropositivity in this context remains somewhat ambiguous [26,44].

Chronic activation of the immune system may contribute to fatigue, as the body expends energy to combat perceived threats [60].

Both emotional and cognitive processes play a role in shaping the perception and consequences of pain, observed in both seropositive and seronegative patients. This highlights an interconnection between pain severity and fatigue, while pain catastrophizing reveals its association with anxiety and fatigue [61].

Localized pain in Sjögren’s syndrome, stemming from inflammation in specific joints or muscles, not only explains the experience of pain but also highlights the correlation between fatigue and low levels of inflammatory markers in the bloodstream, emphasizing that, even with modest elevations in traditional markers, the presence of localized inflammation can contribute to pain and, in turn, to the broader spectrum of fatigue experienced by individuals with Sjögren’s syndrome [2,62].

Research also indicates a connection between fatigue and the quality of sleep. There is a bidirectional relationship between these two, with nighttime disturbances and nocturia being the most important determinants. These factors also contribute to the development of depression and anxiety [26,34]. Fatigue may lead to excessive sleeping, while depression can cause insomnia and disrupted sleep.

Nocturnal respiratory issues in patients with primary Sjögren’s syndrome can contribute to fatigue through various mechanisms, impacting both the quantity and quality of sleep. Conditions like obstructive sleep apnea [63] or interstitial lung disease [64] can disturb normal sleep patterns, causing frequent awakenings to address breathing difficulties. This interruption prevents individuals from attaining deep, restorative sleep, ultimately compromising the overall quality of sleep and contributing to daytime fatigue. Also, both conditions can affect the exchange of oxygen and carbon dioxide in the lungs, and determine the activation of various neural, humoral, thrombotic, metabolic, and inflammatory pathways. If oxygen levels drop during the night due to respiratory issues, it can lead to a state of hypoxemia, which may contribute to feelings of fatigue and exhaustion [65,66]. Poor sleep quality due to respiratory issues can result in daytime sleepiness and a greater need for napping. While napping can provide some relief, it may not fully compensate for the disrupted nighttime sleep, leading to persistent fatigue.

Daytime sleepiness presents a significant challenge for individuals with pSS, contributing to heightened fatigue. Moreover, the quality of sleep in pSS patients is often characterized as inefficient, with frequent awakenings and a higher incidence of hypopnea events. This observation suggests the potential value of including assessments for hypopnea and polysomnographic evaluations in the clinical management of pSS patients [34,45].

Chronic sleep disturbances can negatively impact cognitive function, including memory, concentration, and attention. Cognitive deficits can contribute to feelings of mental fatigue and exhaustion [67].

Several studies have established a connection between disease activity and sleep quality, underscoring the link between sleep quality and extra-glandular manifestations such as myalgia and arthralgia. Each of these factors contributes to the overall experience of fatigue [37].

Research exploring the connection between fatigue and its impact on the quality of life has revealed that patients with pSS experience higher fatigue levels in the morning, which persist throughout the day. Studies have found that patients with pSS experience elevated fatigue throughout the day, including both general and physical fatigue. Furthermore, activity levels were found to fluctuate throughout the day. As a result, a correlation has been identified between heightened evening discomfort and increased fatigue the following day among women with pSS [28,30,38].

In Sjögren’s syndrome, multiple factors influence the connection between activity level and fatigue. Those with the condition often grapple with joint pain, muscle discomfort, and dryness-related challenges affecting the eyes and mouth. These issues can limit physical activity, diminishing engagement in daily tasks and contributing to fatigue. Physical activities may heighten fatigue as the body copes with autoimmune processes and the associated energy demands. Dry eyes and mouth can hinder vision, speech, and overall comfort during activities, leading individuals to reduce their participation, impacting overall activity levels. Additionally, compromised sleep quality further magnifies fatigue, affecting enthusiasm for daily activities. The psychological impact of managing a chronic autoimmune condition, including stress and anxiety, contributes to fatigue. Extended periods of reduced physical activity may induce deconditioning, exacerbating fatigue and contributing to an overall decline in activity levels.

Likewise, in the case of women with pSS, their physical activity levels were significantly reduced and their functional capacity was notably lower. This observation is confirmed by evidence indicating a decrease in aerobic capacity, reduced joint mobility in the upper extremities, compromised muscle function, and impaired balance in patients with pSS, compared to in the general population [30,38].

An exploration of the relationship between the physical aspect and fatigue in pSS patients uncovered a decline in their ability to carry out daily activities. Moreover, a correlation was observed between their quality of life and specific physical symptoms like pain and dryness, as well as somatic factors including fatigue and depression [34,39].

Patients with pSS are not solely concerned with the physical aspects of their condition; cognitive function has also been a subject of interest. Depression and chronic pain can significantly complicate the evaluation of cognitive function, as the majority of patients report experiencing memory-related issues [32,44].

These individuals exhibit measurable deficits in global memory, executive functioning, and coping resources. Moreover, associations have been established between physical functioning, bodily pain, general health, vitality, and emotional functioning within the context of daily life activities [34].

## 5. Conclusions

The distinction between physical fatigue and central fatigue has an important role, especially since the relationship between depression and fatigue is correlated with functional disability, which is determined by both pain and dryness in Sjögren’s syndrome. This phenomenon causes reduced physical activity and prolonged periods of deconditioning, coupled with insomnia and daytime sleepiness, ultimately contributing to fatigue. Central fatigue is often associated with sleep disorders, pain, and affective and cognitive alteration. This is related to the phenomenon of sickness behavior, which also relates to helplessness and is mechanistically and causally linked to fatigue and the quality of life, confirming that fatigue was not related to the severity of autoimmune responses.

The mechanisms underlying fatigue are secondary, distinct from primary autoimmune responses, and are initiated by chronic pain and are compounded by mental stress. This stress triggers low-grade inflammation, forming a feedback loop that both perpetuates pain and compromises the quality of life and sleep. Low-grade inflammation is linked to helplessness and insomnia or a lower quality of sleep and life in general.

In summary, this study marks the initial step toward gaining a better understanding of the intricate interplay of the physical, psychosocial, and cognitive factors that influence fatigue among patients with pSS. While the pathophysiological mechanisms remain a subject of ongoing research, the recognition of depression and the implementation of behavioral strategies offers promise for improving the quality of life and well-being of individuals with Sjogren’s syndrome. This review underscores the importance of a multidisciplinary approach in addressing the multifaceted nature of fatigue within this patient population.

## Figures and Tables

**Table 1:** Sets of keywords/combinations of keywords/syntaxes used for the contextual searches and the related numerical results of our search.

	PubMed	PsycINFO
(physical aspect) AND (fatigue) AND (Sjogren’s syndrome OR Sjogren’s disease OR Sicca)	16	0
(psychological aspects) AND (fatigue) AND (Sjogren’s syndrome OR Sjogren’s disease OR Sicca)	8	0
(social aspects) AND (fatigue) AND (Sjogren’s syndrome OR Sjogren’s disease OR Sicca)	10	0
(sleep disturbance) AND (fatigue) AND (Sjogren’s syndrome OR Sjogren’s disease OR Sicca)	21	0
(daily functioning) AND (fatigue) AND (Sjogren’s syndrome OR Sjogren’s disease OR Sicca)	23	0

**Table 2:** Table with titles, authors journals, and related links of selected articles in our systematic literature review.

Article	First Author	Year of Publication	No. of References	No. of Subjects with pSS Included	Control Lot
Prevalence, Severity and Predictors of Fatigue in Primary Sjogren’s Syndrome	Barbara Segal	2008	[18]	94	no
Sleepiness or fatigue? Can we detect treatable causes of tiredness in primary Sjogren’s syndrome?	Lisa Theander	2010	[26]	72	yes
Fatigue in Daily Life in Patients with Primary Sjögren’s Syndrome and Systemic Lupus Erythematosus	G.L.R. Godaert	2002	[27]	28	yes
Physical activity but not sedentary activity is reduced in primary Sjögren’s syndrome	Wan-Fai Ng	2017	[28]	273	yes
Fatigue in primary Sjögren’s syndrome	P.J. Barendregt	1998	[29]	49	yes
Physical Capacity in Women With Primary Sjogren’s Syndrome: A Controlled Study	Britta Strömbeck	2003	[30]	51	yes
Impaired Functional Status in Primary Sjogren’s Syndrome	Katie L. Hackett	2012	[31]	69	yes
Primary Sjogren’s Syndrome: Cognitive Symptoms, Mood and Cognitive Performance	Barbara Segal	2012	[32]	39	yes
The impact of Sjogren’s syndrome on the quality of sexual life of female patients in the UK: a controlled analysis	Minan Al-Ezzi	2020	[33]	65	yes
The interplay between cognition, depression, anxiety, and sleep in primary Sjogren’s syndrome patients	Radjiv Goulabchand	2022	[34]	32	no
Correlation of sleep quality with fatigue and disease activity among patients with primary Sjögren’s syndrome: a cross-sectional study	Luciana Paula Dardin	2020	[35]	50	no
Sleep quality in patients with primary Sjögren’s syndrome	Roberta Priori	2016	[36]	29	yes
Impact of sleep quality on clinical features of primary Sjögren’s syndrome	Sang Wan Chung	2019	[37]	115	no
Daytime Patterning of Fatigue and Its Associations with the Previous Night’s Discomfort and Poor Sleep Among Women with Primary Sjögren’s Syndrome or Rheumatoid Arthritis	Claire Goodchild	2010	[38]	39	yes
Variability of fatigue during the day in patients with primary Sjögren’s syndrome, systemic lupus erythematosus, and rheumatoid arthritis	Marijn L. Van Oers	2010	[39]	29	yes
Cognition, depression, fatigue, and quality of life in primary Sjögren’s syndrome: correlations	Belgin Koçer	2016	[40]	32	yes
The Effects of Noninvasive Vagus Nerve Stimulation on Fatigue and Immune Responses in Patients With Primary Sjögren’s Syndrome	Jessica Tarn	2018	[41]	50	no
Health-related utility values of patients with primary Sjögren’s syndrome and its predictors	Dennis Lendrem	2013	[42]	639	no
Serum dehydroepiandrosterone sulphate levels and laboratory and clinical parameters indicating expression of disease are not associated with fatigue, well-being and functioning in patients with primary Sjögren’s syndrome	André Hartkamp	2011	[43]	60	yes

**Table 3:** Condensed summary that encompasses the primary fatigue assessment scales.

Scale	Authors of Studies
Fatigue Severity Scale (FSS)	Barbara Segal et al. [44]
	Barbara Segal et al. [32]
	Sang Wan Chung et al. [37]
	Belgin Koçer et al. [40]
Profile of Fatigue (ProF)	Barbara Segal et al. [44]
	Lisa Theander et al. [26]
	Wan-Fai Ng et al. [28]
	Katie L. Hackett et al. [6]
	Barbara Segal et al. [32]
	Claire Goodchild et al. [38]
	Jessica Tarn et al. [41]
	Dennis Lendrem et al. [42]
ESSPRI	Wan-Fai Ng et al. [28]
	Katie L. Hackett et al. [6]
	Radjiv Goulabchand et al. [34]
	Luciana Paula Dardin et al. [35]
	Jessica Tarn et al. [41]
	Dennis Lendrem et al. [42]
VAS fatigue	Lisa Theander et al. [26]
	Wan-Fai Ng et al. [28]
	Minan Al-Ezzi et al. [50]
	Luciana Paula Dardin et al. [35]
Five-dimensional multidimensional fatigue inventory (MFI)	G.L.R. Godaert et al. [27]
	P.J. Barendregt et al. [29]
	André Hartkamp et al. [43]
	Radjiv Goulabchand et al. [34]
	André Hartkamp et al. [43]
	Marijn L Van Oers et al. [39]
Chalder Fatigue Scale (CFS)	Britta Strömbeck et al. [30]
	Radjiv Goulabchand et al. [34]
PROFAD-SSI-SF	Luciana Paula Dardin et al. [35]
FACIT-F	Roberta Priori et al. [36]

**Table 4:** Scales for measuring cognitive dysfunction, depression, anxiety, and sleep disturbance in patients with pSS used in the analyzed publications.

Type of Symptom	Scale	Authors of Studies
Depression	Beck Depression Inventory version II (BDI)	Radjiv Goulabchand et al. [34]
		Sang Wan Chung et al. [37]
	Centers for Epidemiologic Studies Depression scale (CES-D)	Barbara Segal et al. [44]
		Barbara Segal et al. [32]
	Hospital Depression Scale (HADS-D)	Lisa Theander et al. [26]
		Wan-Fai Ng et al. [28]
		Britta Strömbeck et al. [30]
		Katie L. Hackett et al. [6]
		Minan Al-Ezzie et al. [50]
		Roberta Priori et al. [36]
		Jessica Tarn et al. [41]
		Dennis Lendrem et al. [42]
	Zung self-rating scale of depressive symptoms	G.L.R. Godaert et al. [27]
		P.J. Barendregt et al. [29]
		André Hartkamp et al. [43]
	Hamilton Depression Scale	Belgin Koçer et al. [40]
	Beck Depression Inventory	Belgin Koçer et al. [40]
Anhedonia	Chapman’s scale	Radjiv Goulabchand et al. [34]
Anxiety	State Trait Anxiety Inventory (STAI)	Radjiv Goulabchand et al. [34]
	Hospital Anxiety Scale (HADS-A)	Lisa Theander et al. [26]
		Wan-Fai Ng et al. [28]
		Britta Strömbeck et al. [30]
		Katie L. Hackett et al. [6]
		Minan Al-Ezzie et al. [50]
		Roberta Priori et al. [36]
		Jessica Tarn et al. [41]
		Dennis Lendrem et al. [42]
Learned helplessness	Rheumatology Attitude Index	Barbara Segal et al. [44]
Daytime Sleepiness	Epworth Sleepiness Scale (ESS)	Lisa Theander et al. [26]
		Wan-Fai Ng et al. [28]
Sleep disturbance	Lund University Sleep Assessment Questionnaire	Lisa Theander et al. [26]
	Berlin questionnaire	Radjiv Goulabchand et al. [34]
	Epworth Sleepiness Scale (ESS)	Radjiv Goulabchand et al. [34]
		Jessica Tarn et al. [41]
		Dennis Lendrem et al. [42]
	Insomnia Severity Index (ISI)	Radjiv Goulabchand et al. [34]
	Pittsburgh sleep quality index (PSQI)	Luciana Paula Dardin et al. [35]
		Roberta Priori et al. [36]
Restless legs syndrome	Internationally accepted questionnaire for RLS	Lisa Theander et al. [26]
Cognitive domain–executive function	Stroop Interference Test (Stroop)	Barbara Segal et al. [32]
	Trail Making Test B	
	The Wisconsin Card Sorting Test (WCST)	
Cognitive domain–verbal memory and learning	Hopkins Verbal Learning Test Revised (HVLT-R)	Barbara Segal et al. [32]
Cognitive efficiency	The Controlled Oral Word Association test (COWAT)	Barbara Segal et al. [32]
	Trail Making Test B	
	Digit Symbol	

## Data Availability

Please contact the corresponding author for any inquiries regarding data access.
